# Dynamic monitoring of urban built-up object expansion trajectories in Karachi, Pakistan with time series images and the LandTrendr algorithm

**DOI:** 10.1038/s41598-021-02565-9

**Published:** 2021-11-30

**Authors:** Xinrong Yan, Juanle Wang

**Affiliations:** 1 State Key Laboratory of Resources and Environmental Information System, Institute of Geographic Sciences and Natural Resources Research, Chinese Academy of Sciences, Beijing, 100101 China; 2 University of Chinese Academy of Sciences, Beijing, 100049 China; 3 China-Pakistan Earth Science Research Center, Islamabad, 45320 Pakistan; 4 Jiangsu Center for Collaborative Innovation in Geographical Information Resource Development and Application, Nanjing, 210023 China

**Keywords:** Sustainability, Urban ecology

## Abstract

In the complex process of urbanization, retrieving its dynamic expansion trajectories with an efficient method is challenging, especially for urban regions that are not clearly distinguished from the surroundings in arid regions. In this study, we propose a framework for extracting spatiotemporal change information on urban disturbances. First, the urban built-up object areas in 2000 and 2020 were obtained using object-oriented segmentation method. Second, we applied LandTrendr (LT) algorithm and multiple bands/indices to extract annual spatiotemporal information. This process was implemented effectively with the support of the cloud computing platform of Earth Observation big data. The overall accuracy of time information extraction, the kappa coefficient, and average detection error were 83.76%, 0.79, and 0.57 a, respectively. These results show that Karachi expanded continuously during 2000–2020, with an average annual growth rate of 4.7%. However, this expansion was not spatiotemporally balanced. The coastal area developed quickly within a shorter duration, whereas the main newly added urban regions locate in the northern and eastern inland areas. This study demonstrated an effective framework for extract the dynamic spatiotemporal change information of urban built-up objects and substantially eliminate the salt-and-pepper effect based on pixel detection. Methods used in our study are of general promotion significance in the monitoring of other disturbances caused by natural or human activities.

## Introduction

Urban expansion is one of the influences of human social and economic development, and it usually attracts a large concentration of population, resources, and energy^1,2^. From 2001 to 2018, the total area of global cities increased by 1.68 times to 802,233 km^2^^3^. Although the total urban area accounts for only 0.54% of the land surface area^3^, previous studies have shown that rapid urbanization has introduced problems, such as changes in the local climate, fragmented habitats, degradation of land ecological function, and reduction of arable land, forests, and grasslands^2,4^. In particular, urban expansion in some countries and regions has taken up large areas of high-quality arable land and grassland, which also hinders the achievement of the second Sustainable Development Goal (SDG 2, Zero Hunger) established by the United Nations^5,6^. Therefore, continuous monitoring of urban expansion is of great significance in understanding the process of urbanization, analyzing the impact of urbanization on the ecological environment, and planning future cities. The timely change mapping of urban expansion is very important for decision-making for the adjustment of urban management policies and the planning of urban functional layouts.

Traditional methods for monitoring urban expansion are urban land use/cover classification and mapping, which provide important spatiotemporal insights for understanding urban expansion at a regional scale^7,8^. Classification and mapping methods are either based on post-classification comparisons, or a pixel-to-pixel approach for the simultaneous analysis of multispectral patterns in two or more time-series images^9^. Owing to the lack of remote sensing image resources, time-consuming processes, and limited computing capabilities^10^, these methods are mainly used for urbanization monitoring in 5 or 10 years period, and it is difficult to show the dynamic changes on a yearly scale. At the same time, these monitoring methods are based on the interpretation of multi-phase remote sensing images, with extensive manual interpretation and classification, which may lead to the accumulation and propagation of classification errors^11,12^.

With the development of remote sensing technology, time-series imagery combined with Earth Observation (EO) big data cloud computing platform can effectively solve these problems and achieve efficient and low-cost continuous monitoring of urban expansion. EO big data have been accumulating for nearly 50 years since the implementation of several EO projects in the 1970s. The United States Geological Survey has developed research-quality, application-ready science products derived from original Landsat data^13^, which can be used to monitor, assess, and analyze urban expansion on the Google Earth Engine (GEE) platform. GEE is a representative remote sensing cloud computing platform that allows users to implement EO big data storage, management, and spatial analysis using Google infrastructure^14^. These advantages of cloud-based platforms provide an opportunity to monitor quickly and efficiently and continually land disturbances associated with urban expansion.

The change detection algorithm is a state-of-the-art method used for continuous urban expansion. It includes mutation detection and gradient detection algorithm. Mutation detection algorithms includes sub-annual change detection^15^, image trends from regression analysis^16^, exponentially weighted moving average change detection^17^, vegetation change tracker^18^, breaks for additive seasonal and trend^19^, and vegetation regeneration and disturbance estimates over time^20^, which are often used to monitor forest fires, deforestation, and seasonal floods. The gradient detection algorithm include continuous change detection and classification^21^, continuous monitoring of land disturbance^22^, and LT (LandTrendr) algorithm^23^, which are frequently used in scenes of gradual change, such as land degradation and urban expansion. Among them, the LT algorithm can analyze the gain or loss trend of discrete points and shows an excellent performance for extracting the spatial and temporal information of urban expansion^23^. However, LT still faces two challenges in the extraction of spatiotemporal information of urban expansion: (1) Most of the land cover types around cities in arid areas are bare land. The similar spectral and texture information makes urban expansion monitoring more difficult; (2) LT change detection algorithms are pixel-based and produce a salt-and-pepper effect in the results of detecting spatiotemporal information^24^. The salt-and-pepper effect is an uncertain and random phenomenon in the processing process. Isolated pixels with high local spatial heterogeneity between adjacent pixels appear in the results. They are considered to be noise that affects the accuracy and visibility of classification results^25^. The traditional processing method is spatial filtering^26^, but this simple processing increases the possibility of loss of important information^27^.

Therefore, this study aims to propose a new monitoring framework to achieve the following objectives: (1) To studying a method suitable for extracting urban expansion information in arid areas based on the GEE platform and LT algorithm, combined with multiple indices/bands. (2) To eliminating the widespread phenomenon of salt-and-pepper in previous studies. (3) To using this research to provide a method for the continuous monitoring of urban expansion and even other natural or man-made induced interference.

## Results

### Object extraction results of newly urban built-up areas from 2000 to 2020

**Figure 1:**
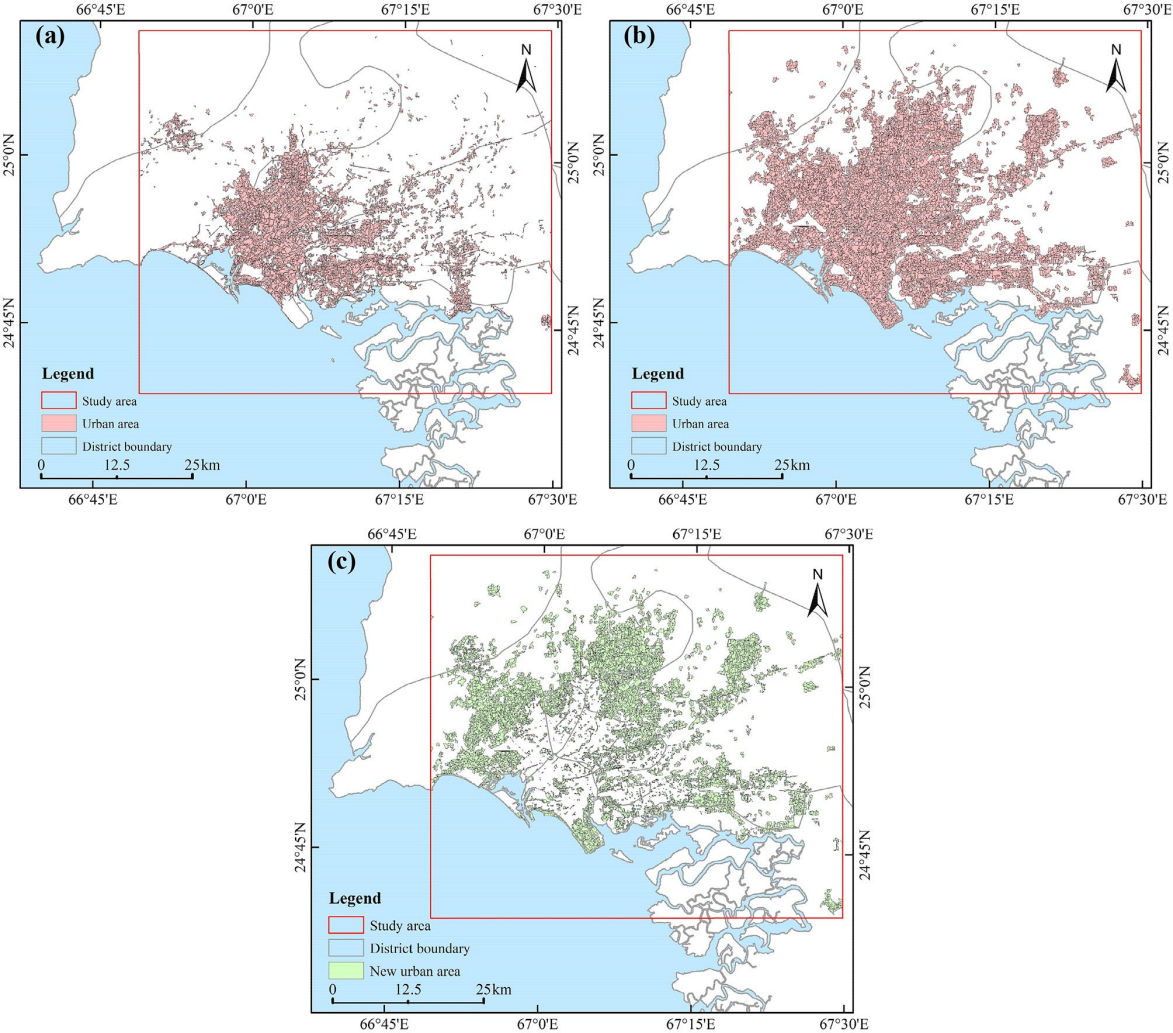
**(a)** Urban areas in 2000; **(b)** urban areas in 2020; **(c)** new urban areas extracted using the object-oriented method (2000–2020). The map created in ESRI ArcMap 10.2 (https://support.esri.com/zh-cn/products/desktop/arcgis-desktop/arcmap/10-2-2).

The urban built-up areas in 2000 and 2020 were obtained using object-oriented segmentation and setting classification rules. Figure 1 shows the spatial patterns of a total of 3113 and 6895 urban built-up objects from the years 2000 and 2020, covering 456.08 km^2^ and 1117.29 km^2^, respectively. We use the “Erase tool” to remove the 2000 results from the 2020 results in ArcMap, and obtain the changes in Karachi from 2000 to 2020. We observed that there were 3782 new urban built-up areas in the span of 21 years, covering an area of 661.21 km^2^ (Fig. 1c).

### Historical change trajectory of urban expansion

#### Annual urban changes analysis

**Figure 2:**
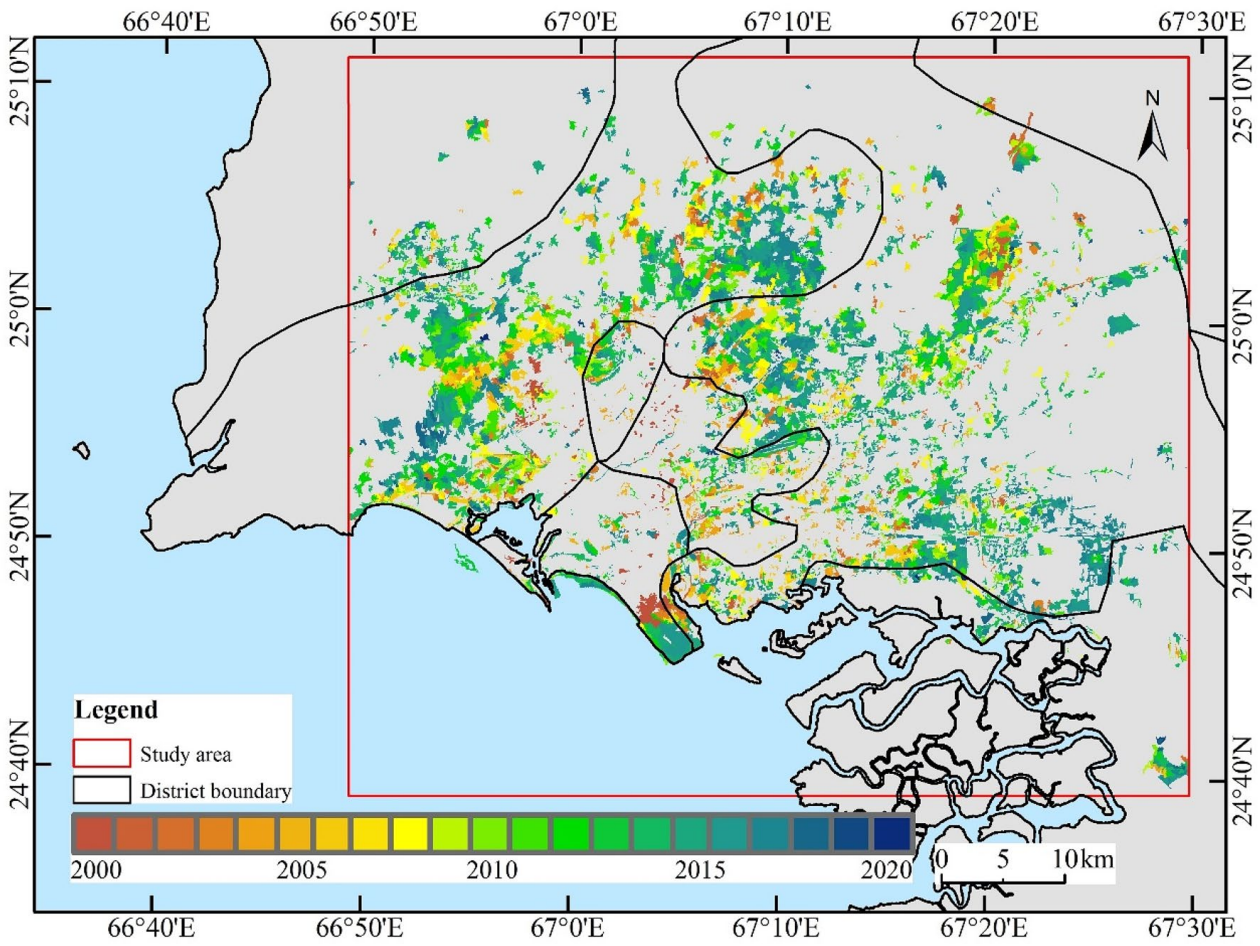
Interannual variation of urban expansion in Karachi. The map created in ESRI ArcMap 10.2 (https://support.esri.com/zh-cn/products/desktop/arcgis-desktop/arcmap/10-2-2).

Figure 2 shows the urban annual change trajectory of Karachi from 2000 to 2020. The spatial and temporal distribution of urban change over these 21 years is shown using gradient mapping for the initial year of monitoring the built-up area. During these 21 years, the newly developed areas were widely distributed around the city center of Karachi, mainly concentrated in the northwestern, northern, northeastern, and eastern areas. The reclamation of the Karachi Port was also one of the main components of the new built-up areas (Fig. 2). Since 2000, the area of urban built-up areas has been increasing annually, with the peak of interannual variation occurring from 2014 to 2017, mainly due to the construction activities in the northern part of the city and along the Karachi coast. The annual growth rate of urban built-up areas ranged from 0.1 to 8.7%, with an average annual growth rate of 4.7% (Table 1).

**Table 1:** Interannual variation area, growth rate, and proportion of urban built-up area in Karachi.

Year	Area (km^2^)	Growth rate of the built-up area (%)	Percentage (%)
2000	11.91	–	1.80
2001	11.75	2.51	1.78
2002	12.61	2.63	1.91
2003	15.22	3.09	2.30
2004	19.65	3.87	2.97
2005	21.25	4.03	3.21
2006	25.07	4.57	3.79
2007	26.06	4.54	3.94
2008	28.99	4.83	4.38
2009	26.89	4.28	4.06
2010	30.90	4.71	4.67
2011	32.54	4.74	4.92
2012	43.16	6.00	6.52
2013	48.03	6.30	7.26
2014	60.57	7.48	9.15
2015	76.25	8.76	11.52
2016	79.65	8.41	12.04
2017	80.72	7.86	12.20
2018	8.68	0.78	1.31
2019	1.23	0.11	0.19
2020	0.57	0.05	0.09

From the interannual perspective of urban expansion, since 2000, cities have gradually expanded from the central areas to the outside, and the urban area has been expanding over time. From 2000 to 2005, urban expansion areas were located in and around the Karachi city center, mainly the undeveloped areas within the city center and the surrounding areas. From 2005 to 2010, the city gradually began to expand to further areas, and the construction activities of satellite cities also increased rapidly around the city. Since 2010, urban expansion has reached an unprecedented speed, with a large amount of idle land in the west, north, and east being developed and gradually expanding outward in spatial and temporal terms.

#### Analysis of duration of urban built-up areas

**Figure 3:**
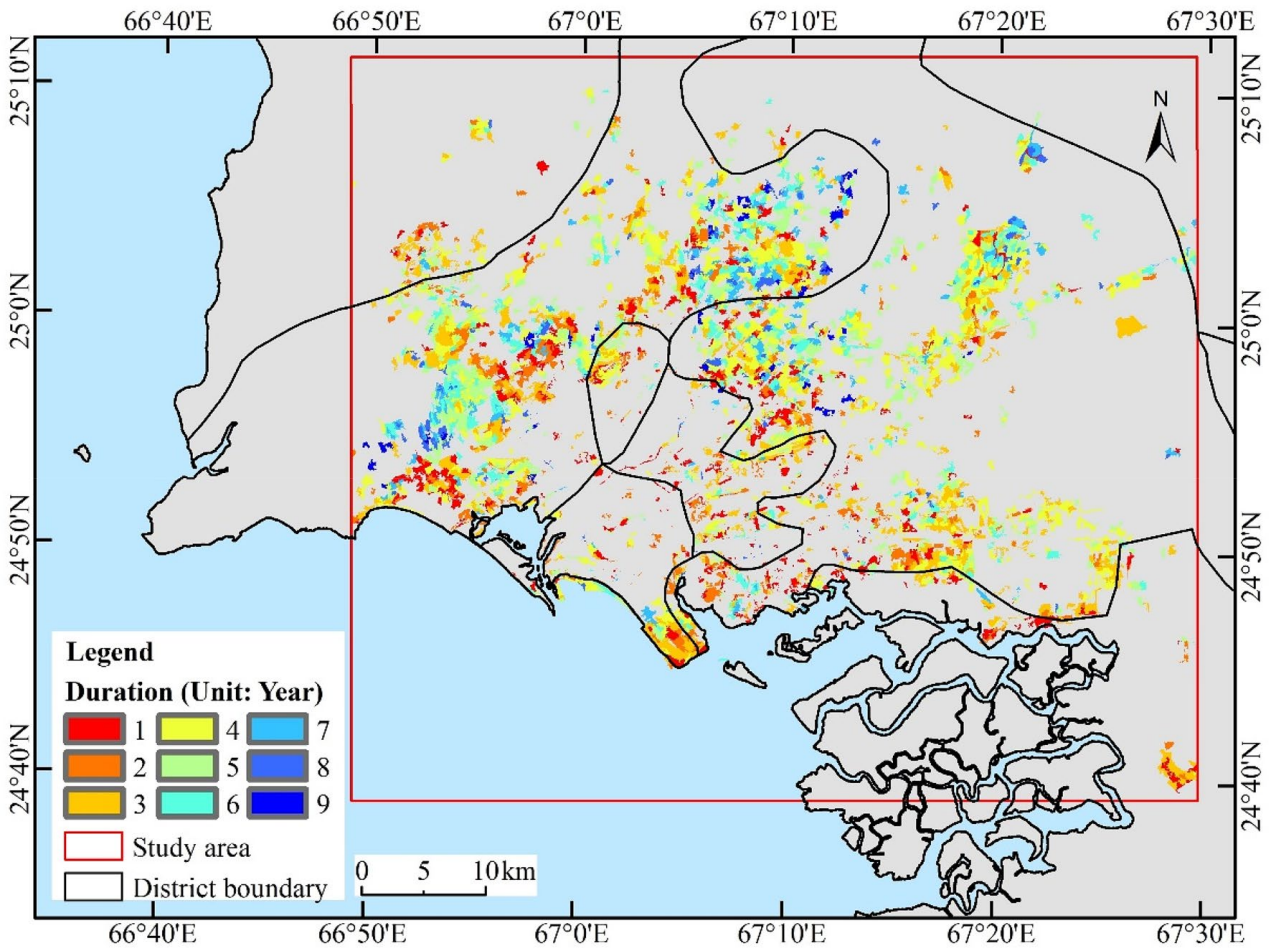
Duration of disturbance of newly added objects in urban built-up areas of Karachi. The map created in ESRI ArcMap 10.2 (https://support.esri.com/zh-cn/products/desktop/arcgis-desktop/arcmap/10-2-2).

**Figure 4:**
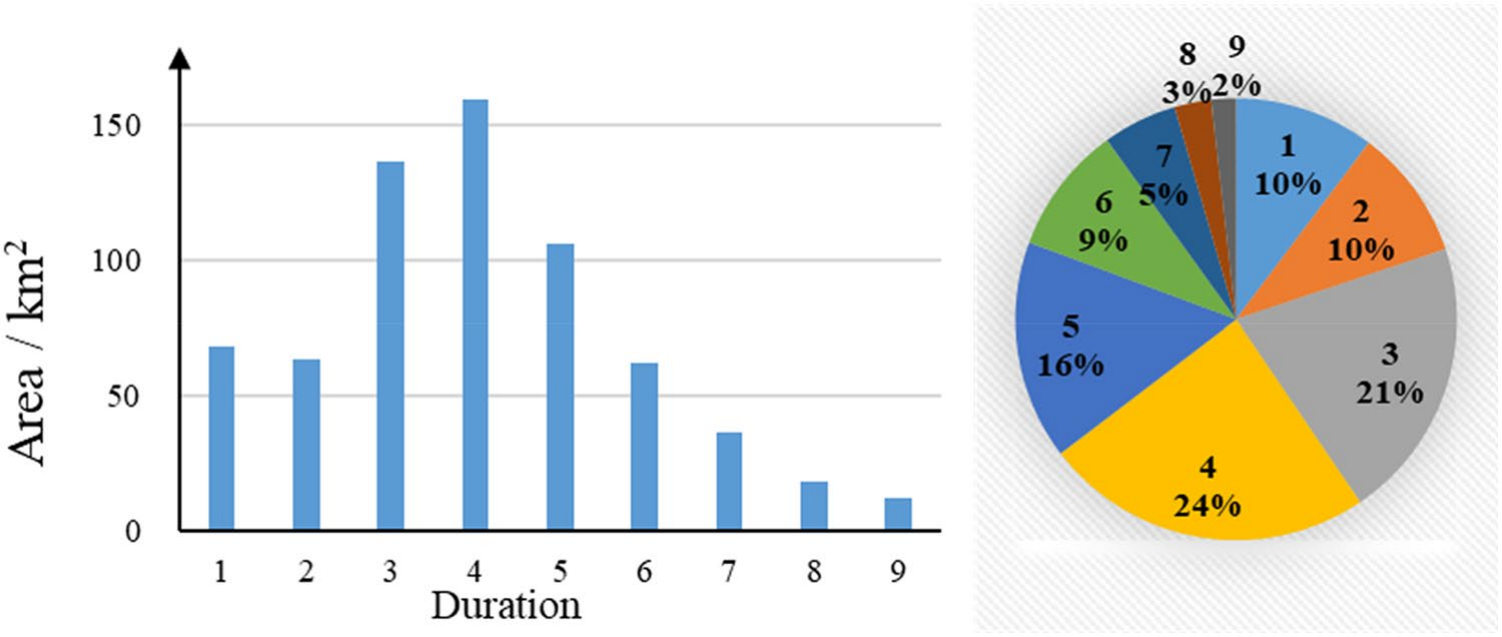
Area of the duration of disturbance of the urban built-up area object.

Figure 3 shows the duration of disturbance of newly developed areas in Karachi from 2000 to 2020, and maps the spatial distribution according to the duration gradient. In general, the duration of disturbance for newly built area objects was 1–9 years. Figure 4 shows the area and proportion of different durations, with the largest proportion being four years, accounting for 16.03% of the total area. Sixty percent of the total area remained undeveloped for less than 5 years. The spatial distribution of the disturbance duration in the urban built-up areas showed the following characteristics: the urban center and coastal areas had a shorter duration of 1–3 years, whereas the western and northeastern areas had a longer duration of 5–8 years. This indicates that the rate of urbanization in the coastal region was higher than that in the other regions.

### Accuracy assessment

#### Accuracy assessment of annual change monitoring

The overall accuracy of the disturbance time of the objects in the urban built-up area was 83.76%, and the kappa coefficient was 0.79. User accuracy ranged from 77.14 to 100%, with an average value of 89%; the lowest value was T3 (2009–2011). The user accuracy was high at T1, T2, and T7. The producer accuracy ranged from 66.67% to 100%, with an average value of 83%, and the lowest value of 66.67% at T7 (2018–2020), while the highest value of 100% was at T1. The error matrix between the prediction year and the reference year can be found in Table S1 online, and the errors of the year are concentrated in the time interval before or after the reference year. The fitting results between the predicted year and reference year from the real sample points on the ground are shown in Fig. S1 online. The results show that the proposed framework can effectively extract the disturbance time of urban built-up areas.

## Discussion

### Spatiotemporal changes and driving factors of urban expansion in Karachi

**Figure 5:**
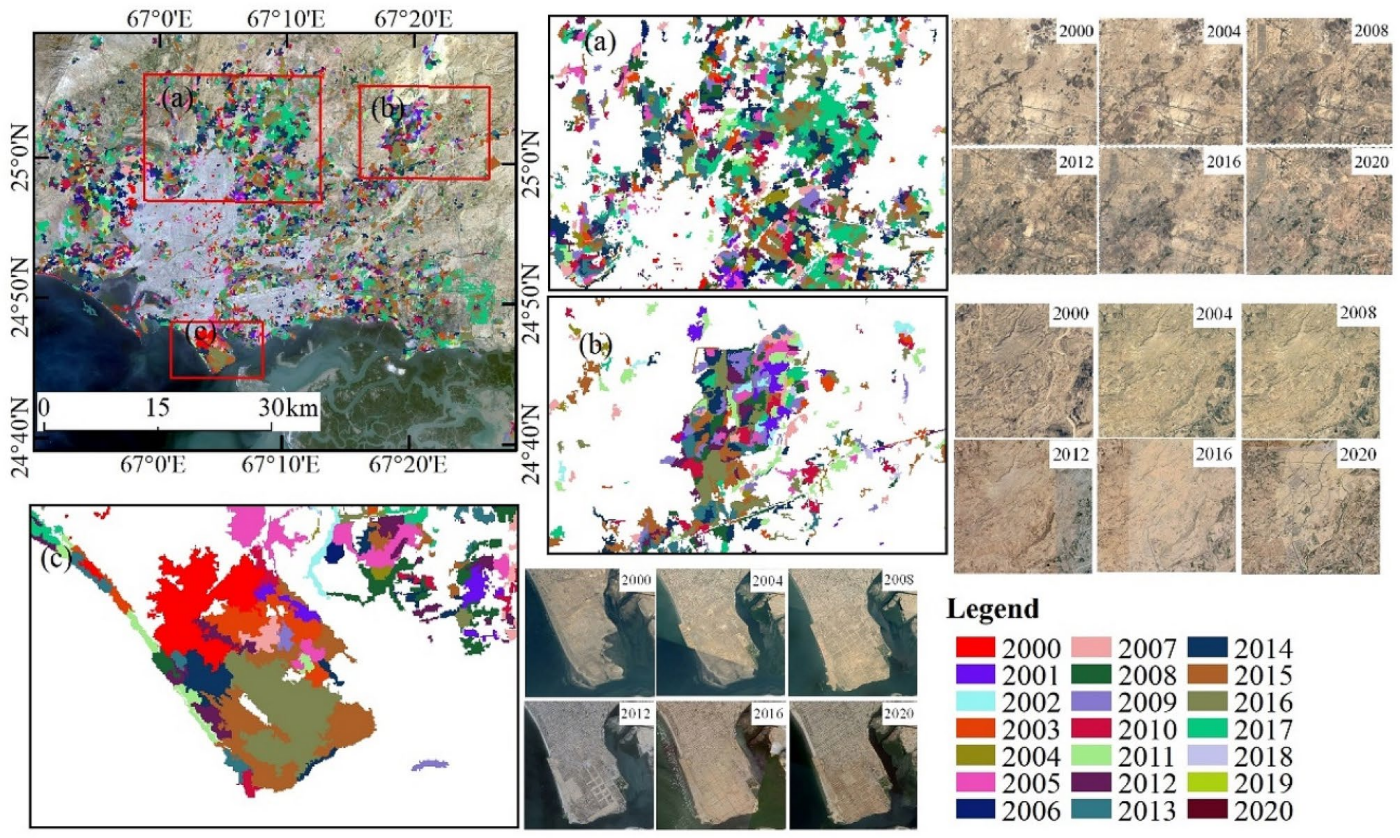
Map of the spatial distribution of new urban expansion areas in Karachi, 2000–2020. **(a)** Newly constructed residence estates in the north, **(b)** newly built theme park and residences in Bahria Town, Karachi, **(c)** newly built residential area to reclaim the sea. (The map created in ESRI ArcMap 10.2, https://support.esri.com/zh-cn/products/desktop/arcgis-desktop/arcmap/10-2-2). Image data source in the upper left: Landsat OLI image (2020) from USGS (United States Geological Survey). Time series images (2000, 2004, 2008, 2012, 2016, and 2020 were obtained from Google Earth Pro 7.3.4 (https://www.google.com/earth/versions/#earth-pro).

Owing to its special geographical location, Karachi has expanded rapidly to inland areas (northwest, north, northeast, and east) and coastal areas (south) over the past 20 years, this is consistent with the findings of previous studies^28,29^. As shown in Fig. 5, new construction sites in the northwest and north, and along the coast are dominated by new residential buildings (Fig. 5a), especially in the Malir and West districts, where the promotion and implementation of the Karachi housing scheme is evident^30^. In terms of the spatial distribution of expansion, we obtained results that are highly consistent with previous studies^29^. The newly added urban built-up areas in the northeast of Karachi have been mainly theme parks and housing projects since 2015, concentrated in the Bahria Town area, covering an area of approximately 35 km^2^ (Fig. 5b). In addition, the newly built areas in the southeast coastal area of Karachi are predominantly housing (Fig. 5c), mainly in the Defence Housing Authority region, with a newly added area of approximately 22 km^2^.

The urban area of Karachi increased by 144.97% from 2000 to 2020. The fastest urban expansion occurred after 2010, accounting for 69.87% of the total area of urban expansion. The peak interannual variation occurred between 2014 and 2017. Population growth and migration are the driving factors of continuous urban expansion^31^. Karachi is the capital of the Sindh province, and its economic and geographical conditions are attractive to the surrounding population. According to the population census data, the population of Karachi was approximately 9.3 million in 1998^32^. In the 2017 census, Karachi had a population of 16.05 million, an increase of 70.9% from 1998, of whom 14.9 million were urban population^32^. The trends of population growth and urban expansion in Karachi are shown in Fig. S2 online. This trend shows that urban areas increase with population size. The results of Baqa et al. (2020) also suggest that population growth in Karachi is one of the main drivers of urban expansion^29^. Karachi’s economic superiority over other Pakistani cities is also an important factor in urban expansion. In 2012, Karachi generated at least 11.4% of GDP, with GDP per capita nearly 44% higher than the national level. Such a prosperous economy has attracted several people and created numerous job opportunities, thus requiring more urban space.

### Combination of object-oriented segmentation method and spectral-temporal segmentation algorithm

In the monitoring framework proposed in this article, the selection of the band/index and the overcoming of the salt-and-pepper effect are key in extracting the information of urban spatial and temporal expansion accurately. Accordingly, normalized burn ratio (NBR), enhanced vegetation index (EVI), and normalized difference vegetation index (NDVI) are often used independently as fitting indices in the parameter input of LT for the extraction of the long-term spatiotemporal change information of forests and other vegetation types^33–35^. This is because the trend change (gain or loss) of a certain band / index is consistent when vegetation are cut down or disturbed. However, for urban expansion monitoring, many factors will affect the changes in these bands/indices, such as the surrounding urban background (for instance, land use type) and building materials. In contrast the disturbance of vegetation, the band/index for monitoring urban expansion has a fixed trend. Online visual analysis shows that using a single band/index does not accurately reflect the spatial and temporal information of urban expansion (https://emaprlab.users.earthengine.app/view/lt-gee-pixel-time-series). Seven bands/indices were used to obtain the urban change information, and the majority value in the information is taken as the result. This is similar to the majority vote method^28^, which can effectively overcome the uncertainty in extracting urban expansion information from a single band/index.

**Figure 6:**
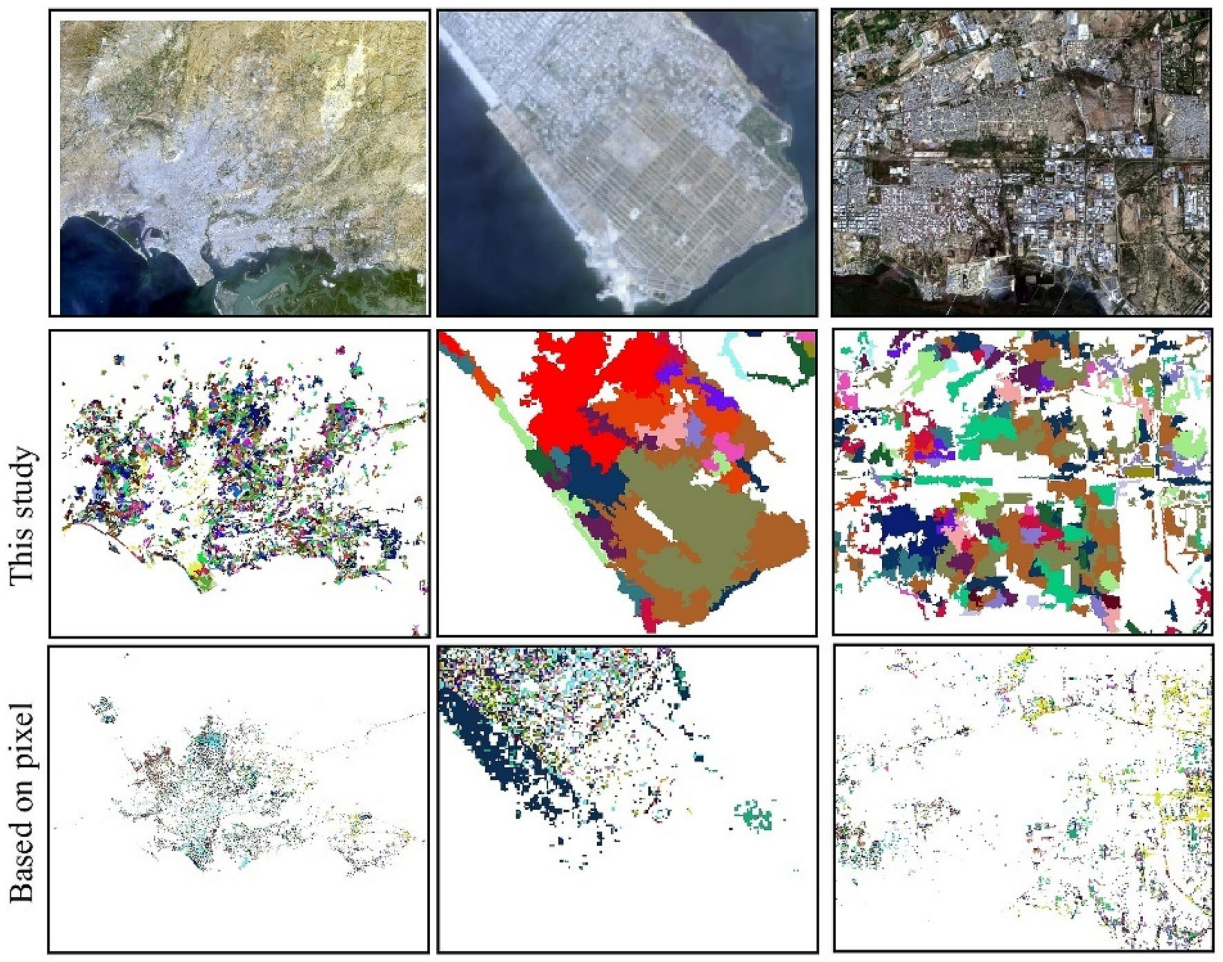
Spatiotemporal information extracted in this study compared with the pixel-based results. (The map created in ESRI ArcMap 10.2, https://support.esri.com/zh-cn/products/desktop/arcgis-desktop/arcmap/10-2-2). Image data source in the top: Landsat OLI image (2020) from USGS.

The salt-and-pepper effect has been widely presented in previous pixel-based studies. For example, more than five pixels (0.045 km^2^) constitute the basic unit of an urban built-up area object, which has often been ignored in previous urban change information extraction^28^. Urban units composed of less than five pixels form a heterogeneous region called “salt-and-pepper”, which will cause some errors in the analysis of urban expansion results. Our extraction process is different from that in previous studies that considered pixels as granularity^36,37^. In contrast, we combined the object-oriented method and adjacent pixels with similar spectra and textures into the research unit. As shown in Fig. 6, the results of the spatial and temporal expansion information of Karachi obtained in this study were compared with the results based on pixel research^38^. It was demonstrated that considering multiple pixels as a collection can effectively overcome the salt-and-pepper pixels in the extraction results.

### Comparisons with open-access products

**Figure 7:**
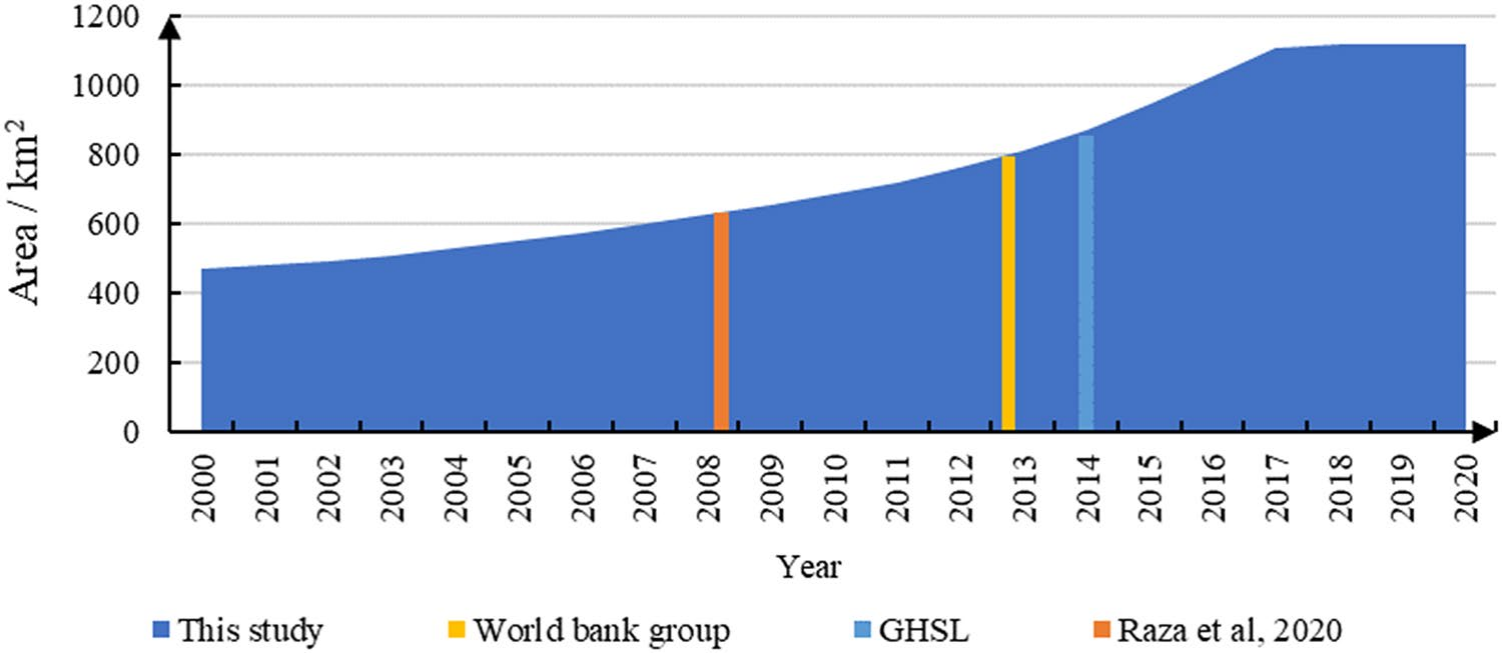
Urban area of Karachi estimated using this study and four open-access products.

The results of this study were compared with those of open-access products (Fig. 7) to determine the differences in area estimation. Open-access products include the Global Human Settlement Layer (GHSL) in 2014^39^, World Bank data in 2013^30^, and the study by Raza et al. in 2008^40^. The urban built-up area of all the three sources, in three different years, closely matched our results, with differences of 17.61 km^2^, 14.11 km^2^, and 2.41 km^2^, accounting for 2.02%, 1.74%, and 0.38% of the total area in this study, respectively. This estimation area comparison is not considered as the ground truth but is included to provide a reference for the accuracy evaluation of different data sources.

**Figure 8:**
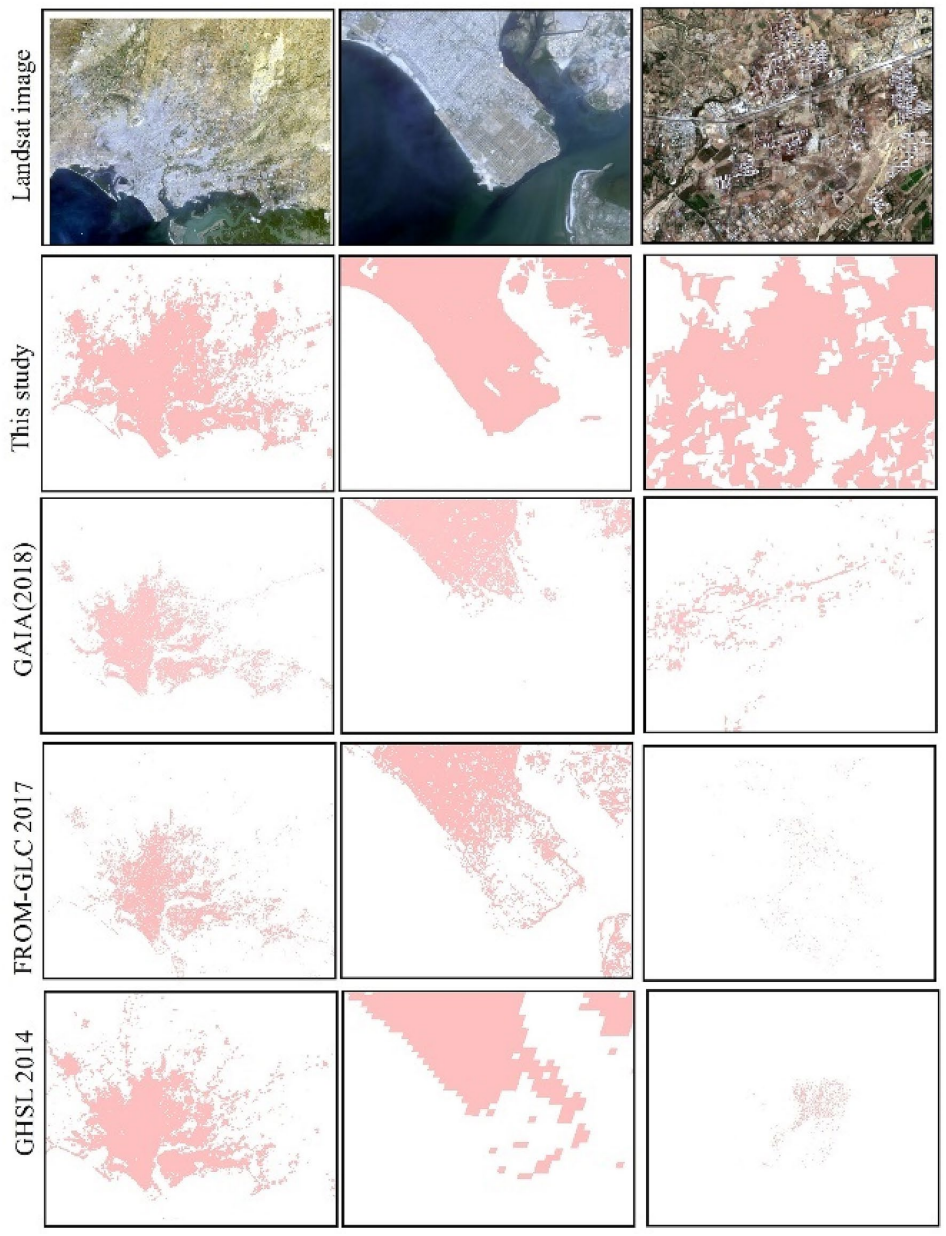
Comparison among this study, GAIA, FROM-GLC, and GHSL. Landsat OLI images from 2020 are shown in red, green, and blue (the map created in ESRI ArcMap 10.2, https://support.esri.com/zh-cn/products/desktop/arcgis-desktop/arcmap/10-2-2).

Figure 8 shows the spatial comparison of this study, Global Artificial Impervious Area (GAIA), GHSL, and Finer Resolution Observation and Monitoring of Global Land Cover (FROM-GLC)^41^. Differences between these products can be observed due to several factors, such as temporal and spatial scales, methods, and data processing. As with GHSL, more satellite town areas and built-up areas around bare land are captured. However, in contrast to GHSL, the footprint data of urban expansion in Karachi were generated at annual intervals, which was more helpful in understanding the characteristics and trends of urban expansion.

### Methodology transferability and limitations

In this study, we propose a new framework for extracting spatiotemporal change information of urban expansion. In the image reconstruction part, cloudless algorithms were used to synthesize time-series images with the support of GEE's powerful computing capabilities. This allows us to select the pixel with the best quality over a period and provide good data support for the extraction of change information of objects in urban areas. This study shows that LT is not only capable of monitoring forest and farmland disturbances but also has great potential in the long-term monitoring of urban regional disturbances. We innovatively propose considering the object of the urban area as the research unit, which can largely eliminate the salt-and-pepper effect of neighboring pixels. The time and place of the disturbed areas of the city for different years can be captured accurately and effectively without increased workload. This also improves the suitability of this method in other regions of the world.

However, urban development is a process of dynamic change, including urban expansion, contraction, reconstruction, and other processes, which are related to several factors, such as policymaking, population growth, and economic development. The proposed monitoring framework is effective for the expansion monitoring of most cities in developing countries because the changes in these cities are dominated by expansion. However, our framework is not yet fully capable of monitoring changes in cities of most developed countries where urban shrinkage is a major process of urban change (such as the Rust Belt in the U.S.). In future studies, we can use remote sensing technology to further monitor multiple processes of urbanization, including both expansion and shrinkage.

## Conclusions

Based on the remote sensing cloud platform of GEE, we propose a framework for object change detection in urban built-up areas that combines an object-oriented segmentation method and a spectral-temporal segmentation algorithm. This framework efficiently extracted the spatiotemporal information of urban expansion using seven bands/indices in Karachi, located in an arid region, and avoided salt-and-pepper noise based on pixel detection. The results showed that Karachi has maintained continuous expansion over the past 21 years, with an average annual growth rate of 4.7%. The spatial distribution of urban expansion is mainly concentrated in the northeast and east inland directions, and it begin mainly after 2010. The duration of construction activity was significantly shorter in coastal areas (1–3 years) and city centers than in inland areas (5–8 years). The framework can discover the detailed urban surface disturbance regions in Karachi, including the spatial range and time of disturbance of the 3782 newly added urban built-up area objects from 2000 to 2020. This case study provides a new perspective for extracting urban expansion information from long time-series remote sensing images. We hope that our research can support decision makers in understanding the continual temporal and spatial changes in urban areas. Furthermore, the framework can be extended to monitor the spatiotemporal information of other land disturbances caused by natural or human activities.

## Methods

### Study area

**Figure 9:**
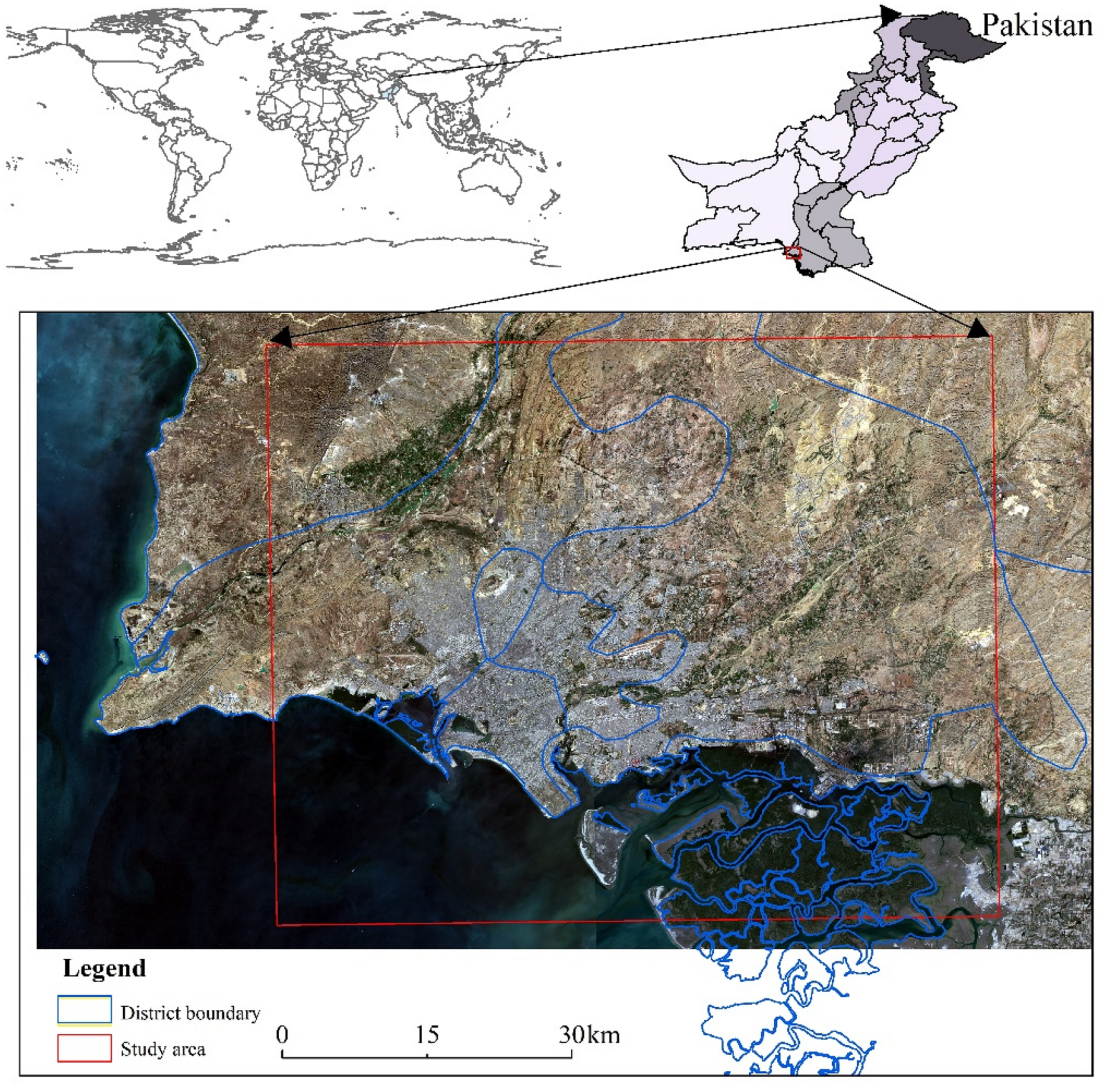
Location of the study area in Karachi, Pakistan. The zoomed-in image (with a red outline) shows the study area (approximately 3527 km^2^) including the urban area of Karachi. The images of Landsat OLI are displayed in true color bands (red, green, and blue). The map created in ESRI ArcMap 10.2, https://support.esri.com/zh-cn/products/desktop/arcgis-desktop/arcmap/10-2-2.

Karachi is the largest city in Pakistan, located of the southern coast on the western edge of the Indus Delta and on the plain between the Leri and Maryr rivers (Fig. 9). It covers an area of 3,527 km^2^ and includes an urban area of 1,821 km^2^. Owing to its special geographical location, Karachi has become a major port in the Indus River Valley. It is an important shipping, rail, and air transportation port in Pakistan. Karachi has a subtropical desert climate, with high temperatures and little rain for most of the year. The average minimum temperature in winter (January and February) was observed to be 13 °C, and the average maximum temperature in summer (May and June) was observed to be 34 °C. Rainfall in Karachi is rare, with an average annual precipitation of only 200 mm. This study focuses on the Karachi urban area, with a geographic range of 66° 42′ 58" E–67° 29′ 58" E, 24° 37′ 16" N–25° 11′ 54" N.

### Overview of the LT segmentation algorithm

LT is a set of spectral-temporal segmentation algorithms based on remote sensing image pixels that are useful for change detection in a time series of moderate-resolution satellite imagery. The spectral time series data based on the trajectory generated by the LT algorithm have almost no interannual signal noise. The LT algorithm uses a time segmentation strategy based on regression and a point-to-point fitting spectral index as a time function, allowing the capture for slow-evolving processes, such as recovery and unexpected events^42^. Interactive Data Language (IDL) initially implemented LT, and later Google engineers ported LT to the GEE platform^14,23^. The GEE framework nearly eliminates the onerous data management and image-preprocessing aspects of IDL implementation. LT combined with GEE also simplifies tedious data management and image preprocessing by directly accessing geospatial datasets in GEE. Thus far, LT has been applied in research on forest resource monitoring^43^, abandoned land identification^44^, land cover change trajectories^45,46^, habitat monitoring^47^, continuous monitoring of land disturbance^48^, and rapid land cover mapping^49^. The LT algorithm has been used to monitor changes in urban areas. The algorithm realizes the change monitoring of urban areas by analyzing the time-spectral trajectory of each pixel. The input for each pixel is the annual time series of one spectral band or index plus the date. The processing procedure for finding the best model involves removing noise-induced spikes (outliers), identifying potential vertices (breakpoints), fitting trajectories, and setting the optimal number of segments^46^.

### Data acquisition and preprocessing

Table 2 shows the data type, description, and sources used in this study. We applied Landsat image data on the GEE platform. The first Landsat satellite was launched in 1972 by the United States Land Program. By 2020, it has included a multispectral scanner, thematic mapper (TM), enhanced thematic mapper (ETM +), and operational land imager (OLI) sensor, which have accumulated a large amount of data for Earth observation^14^. With the support of GEE, we selected all the available surface reflectance data of Karachi from Landsat, including data from Landsat TM, ETM + , and OLI sensors, and removed Landsat 7 ETM + SLC-off images with data gaps. The QA band is used to assess image quality, removing cloud, snow, water and shadow influences. We synthesized the cloudless annual images of Karachi from 2000 to 2020, including blue, green, red, near infrared, shortwave infrared 1, and shortwave infrared 2 bands.

**Table 2:** Data type, description, and source in this study.

Data Type	Description	Period	Source
Landsat image collection (TM/ETM + /OLI)	Annual atmospherically corrected surface reflectance; collection from June to September 2000–2020, for use with LT. Path/row 152/043	2000–2020	Surface reflection from Google Earth Engine
All available high-resolution images	Analysis of change of indices in 2000–2020; used for the validation of LT spatiotemporal segmentation results	2000–2020	Google Earth

**Figure 10:**
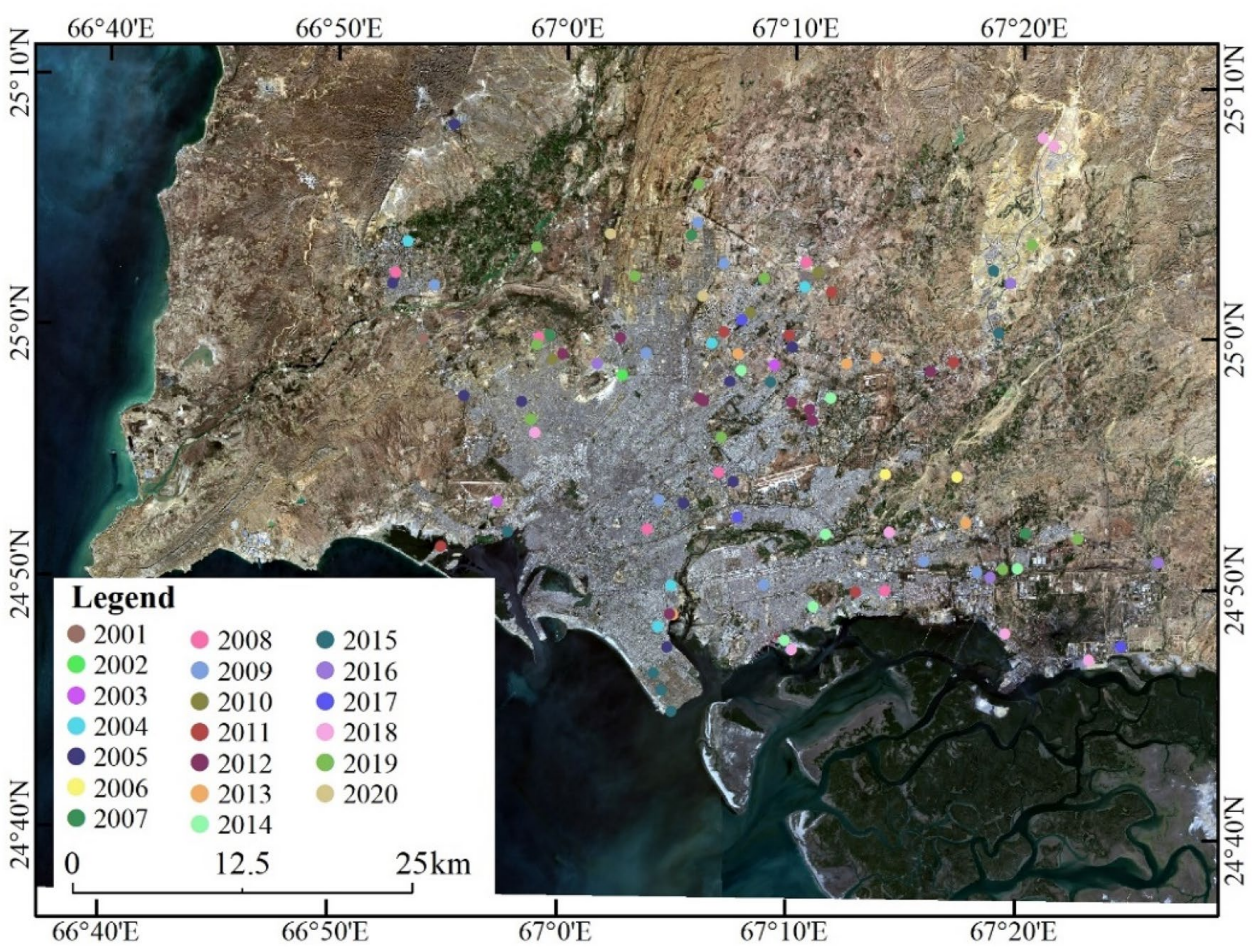
Distribution of ground sample points from 2000 to 2020 (the map created in ESRI ArcMap 10.2, https://support.esri.com/zh-cn/products/desktop/arcgis-desktop/arcmap/10-2-2). Image data source: Landsat OLI image (2020) from USGS.

The ground sample data were obtained from the interpretation of historical high-resolution remote sensing images of Google Earth. Two hundred ground sample points recorded the change year information of urban building activities or disturbances (Fig. 10). The application of sample data to LT segmentation involves the analysis of spectral curves (using 100 samples) and verification of the segmentation results (using 200 samples).

### Extraction of urban expansion information

**Figure 11:**
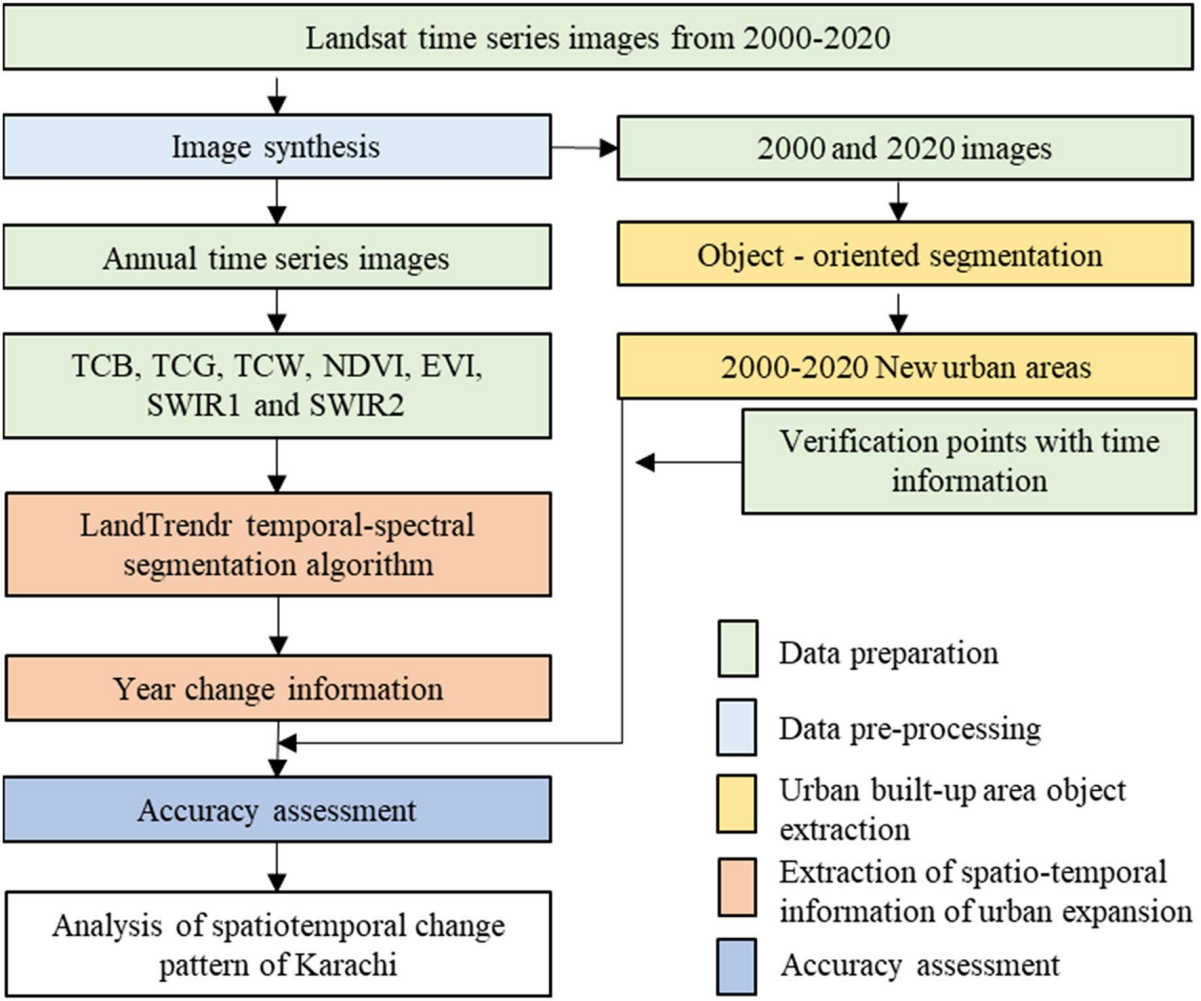
Flowchart of urban expansion monitoring.

**Figure 12:**
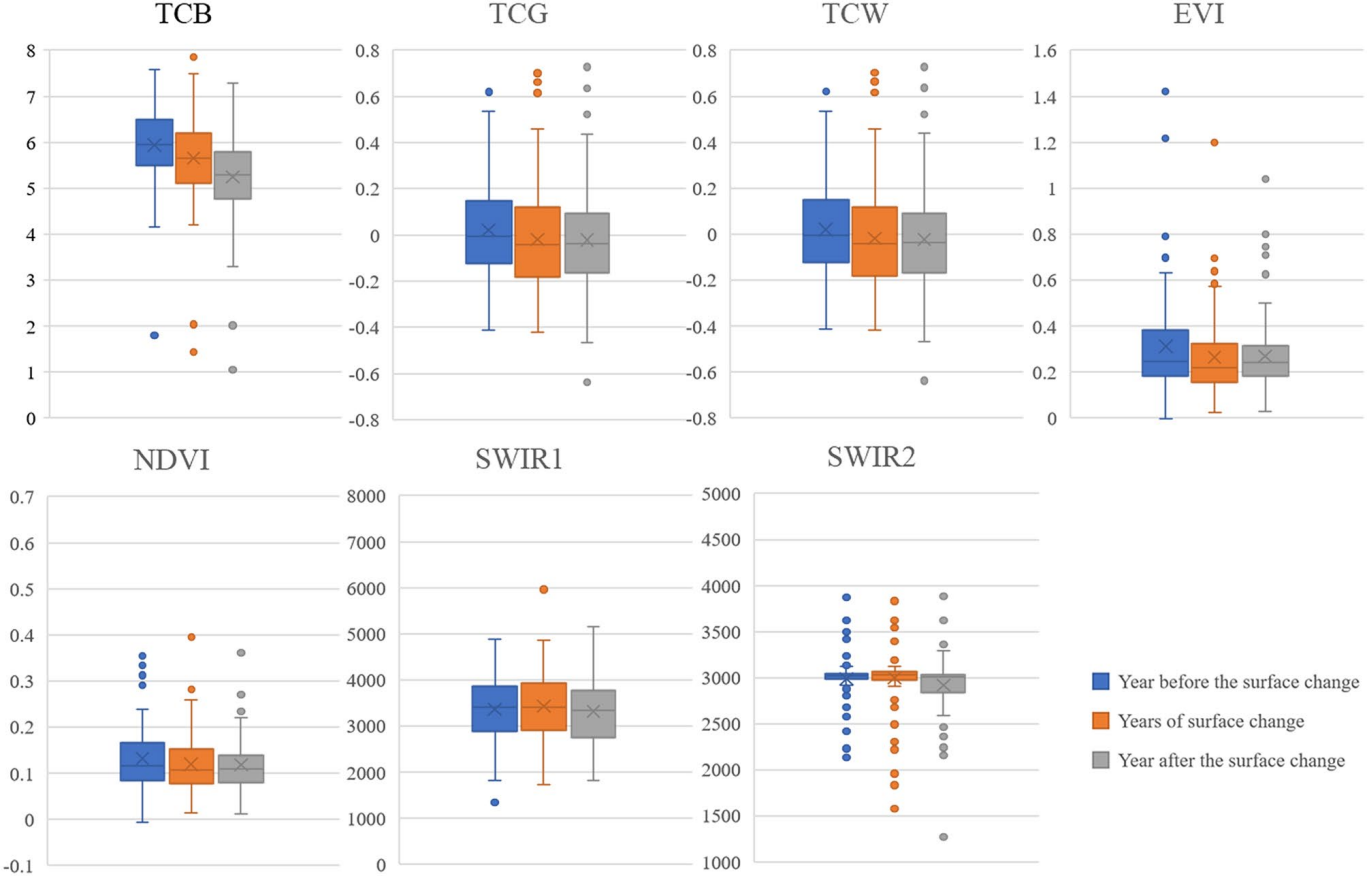
Numerical variation of different bands/indices before, during, and after the disturbance in the built-up area.

As shown in Fig. 11, this study combined the object-oriented segmentation method with the LT spectral-temporal segmentation algorithm to propose a framework for detecting urban expansion, which mainly includes the following:

- (1) Object extraction of built-up areas in Karachi
- First, the objects of urban built-up areas in the initial year (2000) and final year (2020) were generated by using the object-oriented segmentation method. With the support of eCognition software, the segmentation threshold is set with the support of eCognition software to generate objects with uniform attributes. Through segmentation scale optimization, the appropriate segmentation scale is 15. The combination of different bands was used to calculate the NDVI and the Normalized Difference Water Index (NDWI). After setting different thresholds for the two indices (NDVI was 0.05, and NDWI was 0), vegetation and water objects could be detected and then be removed. We use Google Earth’s high-resolution remote sensing images from 2000 to 2020 to manually edit the extracted urban area objects to ensure more accurate results.
- Second, to obtain the object of new urban areas from 2000 to 2020, we used the Erase tool in ArcMap to eliminate the common part of the urban areas in the 2 years.
- Finally, we used the Sentinel-2 and GF-2 images of 2020 to edit the objects in the urban built-up areas manually to ensure the accuracy of the results.
- (2) Spectral-temporal segmentation with LT
- LT requires annual image collection during spectral-temporal segmentation. Annual image collections are usually composed of a time series of bands/indices. The best candidate indices/bands are those that can highlight the changes and capture valuable information about the surface properties of the study area. The choice of indices is based on the LT results of visual inspection, based on the analysis of the LT visual website (https://emaprlab.users.earthengine.app/view/lt-gee-pixel-time-series). We used seven indices/bands that reflect the changes in Karachi’s urban area in the arid region better, namely, TCB, tasseled cap green (TCG), tasseled cap wet (TCW), NDVI, EVI, short-wave infrared 1 (SWIR1), and short-wave infrared 2 (SWIR2). The three features (TCB, TCG, and TCW) of the tasseled cap transform can reduce the data dimension while preserving the multi-band image information^50^. These features have been widely used in urban land-cover mapping in previous studies^51,52^. In addition, a set of parameters to control segmentation is required for spectral-temporal segmentation. To obtain better segmentation results, we analyzed the numerical changes in the seven bands/indices in different years according to the 100 collected ground sample points of the objects of urban built-up. The statistical results show that the seven indices show a downward trend before and after being disturbed (Fig. 12), which is consistent with previous research results^28^. Accordingly, we set the segmentation parameters for LT as shown in Table S2 online to ensure more accurate segmentation results. According to the year information of urban built-up area before, during and after land surface change recorded by sample points, we drew the curves of each index to obtain the optimal LT parameters respectively. The purpose of this study is to track the continuous change of construction land in urban areas, so the indexes curve of bare land is not plotted.
- (3) Monitoring the year and duration information of newly added of urban built-up area with LT
- In the extracted urban built-up area object, each object contained multiple pixels. According to the segmentation results of seven indices using LT method, the majority voting method was used to confirm the year and duration information of objects in urban built-up areas^53^, including the following two steps.
- (1) Determine the year and duration of change of a single pixel. After the segmentation of the seven indices, a single pixel is used as a unit to generate preliminary results according to the method of majority voting;
- (2) Determine the year and duration of change of the objects of urban built-up areas. According to the preliminary results, the main value voting method is adopted to generate the results by taking the object of the urban built-up area as a unit.
- We extract the change information of the year and duration of the urban built-up area objects through the zonal statistics function in ArcMap 10.2.
- (4) Accuracy assessment
- For the urban change trajectory analysis, we used 200 ground verification points to evaluate the accuracy of the change year of the extraction. Because of the time difference between the sampling points on Google Earth and the annual image synthesis time, the real time of surface change is inconsistent with the image time (for example, the image in March is used in the image synthesis, the surface change time occurs in May, and the monitoring result of LT will be the next year). To ensure the accuracy of the verification results further, we divided 2000–2020 into seven periods based on a period of three years by referring to the accuracy assessment method^28,37^ the confusion matrix method was used to verify the extraction accuracy of the changed years.

## Supplementary Information

Supplementary Information.

## Data Availability

The data that supports the findings of this study are available from the corresponding authors upon request.
