# Supplementary material for: Dynamic monitoring of urban built-up object expansion trajectories in Karachi, Pakistan with time series images and the LandTrendr algorithm
Source: Sci Rep. 2021 Nov 30;11:23118. doi: 10.1038/s41598-021-02565-9 (PMC8632927; doi:10.1038/s41598-021-02565-9)
Supplement: Supplementary file 1 — Supplementary Information. [file 41598_2021_2565_MOESM1_ESM.docx]

**Dynamic Monitoring of Urban Built-up Object Expansion Trajectories in Karachi, Pakistan with Time Series Images and the LandTrendr Algorithm**

Xinrong Yan ^1,2^, Juanle Wang^* 1,2,3,4^

1 State Key Laboratory of Resources and Environmental Information System, Institute of Geographic Sciences

and Natural Resources Research, Chinese Academy of Sciences, Beijing 100101, China

2 University of Chinese Academy of Sciences, Beijing 100049, China

3 China-Pakistan Earth Science Research Center, Islamabad 45320, Pakistan

4 Jiangsu Center for Collaborative Innovation in Geographical Information Resource Development and Application, Nanjing 210023, China

* Correspondence: wangjl@igsnrr.ac.cn; Tel.: +86-010-6488-8016

Table S1 online. Accuracy of identifying the year of objects of urban built-up area

|  | Reference Year | | | | | | | |
| --- | --- | --- | --- | --- | --- | --- | --- | --- |
| Prediction Year | T1 | T2 | T3 | T4 | T5 | T6 | T7 | User accuracy |
| T1 | 7 | 0 | 0 | 0 | 0 | 0 | 0 | 100% |
| T2 | 0 | 18 | 0 | 0 | 0 | 0 | 0 | 100% |
| T3 | 0 | 5 | 27 | 3 | 0 | 0 | 0 | 77.14% |
| T4 | 0 | 0 | 3 | 30 | 2 | 0 | 0 | 86% |
| T5 | 0 | 0 | 0 | 4 | 39 | 9 | 0 | 75% |
| T6 | 0 | 0 | 0 | 0 | 5 | 42 | 1 | 87.50% |
| T7 | 0 | 0 | 0 | 0 | 0 | 0 | 2 | 100% |
| Producer accuracy | 100% | 78.26% | 90.00% | 81.08% | 84.78% | 82.35% | 66.67% |  |

T1: 2000–2002; T2: 2003–2005; T3: 2006–2008; T4: 2009–2011; T5: 2012–2014; T6: 2015–2017; T7: 2018–2020.

Table S2 online. Optimal segmentation parameters of the seven indices

| **Parameter** | **Band or index** | | | | | | | |
| --- | --- | --- | --- | --- | --- | --- | --- | --- |
| index | TCB | TCG | TCW | NDVI | NDMI | EVI | B5 | B7 |
| startYear | 1999 | 1999 | 1999 | 1999 | 1999 | 1999 | 1999 | 1999 |
| endYear | 2020 | 2020 | 2020 | 2020 | 2020 | 2020 | 2020 | 2020 |
| startDay | 1101 | 1101 | 1101 | 1101 | 1101 | 1101 | 1101 | 1101 |
| endDay | 1230 | 1230 | 1230 | 1230 | 1230 | 1230 | 1230 | 1230 |
| maxSegments | 10 | 10 | 10 | 10 | 10 | 10 | 10 | 10 |
| spikeThreshold | 0.9 | 0.9 | 0.9 | 0.9 | 0.9 | 0.9 | 0.9 | 0.9 |
| vertexCountOvershoot | 3 | 3 | 3 | 3 | 3 | 3 | 3 | 3 |
| preventOneYearRecovery | TRUE | TRUE | TRUE | TRUE | TRUE | TRUE | TRUE | TRUE |
| pvalThreshold | 0.05 | 0.05 | 0.05 | 0.05 | 0.05 | 0.05 | 0.05 | 0.05 |
| bestModelProportion | 0.75 | 0.75 | 0.75 | 0.75 | 0.75 | 0.75 | 0.75 | 0.75 |
| minObservationsNeeded | 9 | 9 | 9 | 9 | 9 | 9 | 9 | 9 |
| delta | lost | lost | lost | lost | lost | lost | lost | lost |
| sort | greatest | greatest | greatest | greatest | greatest | greatest | greatest | greatest |

Fig. S1 online. Fitting plots of prediction year and reference year obtained from 100 sample sites. Prediction year: Fitting results based on the framework extracted in this study; Reference year: Visual interpretation of the disturbance year by using Google high-resolution remote sensing images of the Earth

Fig. S2 online. Trend of population growth (in millions) in Karachi
